# High-frequency torsional Alfvén waves as an energy source for coronal heating

**DOI:** 10.1038/srep43147

**Published:** 2017-03-03

**Authors:** Abhishek Kumar Srivastava, Juie Shetye, Krzysztof Murawski, John Gerard Doyle, Marco Stangalini, Eamon Scullion, Tom Ray, Dariusz Patryk Wójcik, Bhola N. Dwivedi

**Affiliations:** 1 Department of Physics, Indian Institute of Technology (BHU), Varanasi-221005, India; 2 Armagh Observatory, College Hill, Armagh, BT61 9DG, N. Ireland; 3 Group of Astrophysics, Institute of Physics, UMCS, Lublin, Poland; 4 INAF-OAR National Institute for Astrophysics, 00040, Monte Porzio Catone, RM, Italy; 5 Department of Mathematics & Information Sciences, Northumbria University, Newcastle Upon Tyne, NE1 8ST, UK; 6 Dublin Institute for Advanced Studies, 31 Fitzwilliam Place, Dublin 2, Ireland

## Abstract

The existence of the Sun’s hot atmosphere and the solar wind acceleration continues to be an outstanding problem in solar-astrophysics. Although magnetohydrodynamic (MHD) modes and dissipation of magnetic energy contribute to heating and the mass cycle of the solar atmosphere, yet direct evidence of such processes often generates debate. Ground-based 1-m Swedish Solar Telescope (SST)/CRISP, Hα 6562.8 Å observations reveal, for the first time, the ubiquitous presence of high frequency (~12–42 mHz) torsional motions in thin spicular-type structures in the chromosphere. We detect numerous oscillating flux tubes on 10 June 2014 between 07:17 UT to 08:08 UT in a quiet-Sun field-of-view of 60” × 60” (1” = 725 km). Stringent numerical model shows that these observations resemble torsional Alfvén waves associated with high frequency drivers which contain a huge amount of energy (~10^5^ W m^−2^) in the chromosphere. Even after partial reflection from the transition region, a significant amount of energy (~10^3^ W m^−2^) is transferred onto the overlying corona. We find that oscillating tubes serve as substantial sources of Alfvén wave generation that provide sufficient Poynting flux not only to heat the corona but also to originate the supersonic solar wind.

Continuous generation of radiation and supersonic wind from the Sun’s chromosphere and corona requires a large input of energy (~10^2^–10^4^ W m^−2^) to balance these losses^1^. The role of magnetohydrodynamic (MHD) waves and small-scale magnetic reconnection causing nano-flare heating have been explored as primary candidates to energize the solar atmosphere. However, direct evidence of energy sources and their dissipation are not yet fully understood^2^,^3^,^4^. In the era of high resolution space and ground-based observations, it is now revealed that energy and mass transport in the quiescent solar atmosphere are associated with localized static and flowing flux tubes (e.g., network & inter-network magnetic fields, spicules, vortices etc) possessing various plasma and wave processes^5^,^6^,^7^,^8^. Here, we observe directly, for the first time, the ubiquitous presence of high frequency (~12–42 mHz) torsional oscillations at apparent surfaces composed of thin spicular-type structures rooted in the quiet-Sun magnetic network. These observations are described by torsional Alfvén waves associated with high frequency drivers transferring ~10^3^ W m^−2^ energy into the overlying corona. These oscillating tubes serve as substantial sources of Alfvén wave generation providing sufficient Poynting flux to heat the solar corona and in originating the nascent solar wind.

Quiet-sun magnetic networks are the locations where field lines fan out into the outer atmosphere supporting waves and exotic plasma dynamics^9^,^10^. The magnetic skeleton of the bundle of fine structured small-scale flux tubes becomes visible when remnants of plasma flows (e.g., spicules, jets, surges) are confined within their boundaries. Various other similar structures are prevalent in the solar chromosphere and well resolved with modern day instruments, e.g., on-disk counterparts of type-II spicules, chromospheric counterparts of the transition region network jets, etc.^11^,^12^. A tube with its fine structures, each 120–215 km wide, is observed using CRISP on the Swedish Solar Telescope (see yellow and green expanding cylinders in Fig. 1A.1, respectively). In an integrated view, the tube’s projected height and width at the top are respectively ~4 Mm and ~1.5 Mm. Using visualization software, we measure the structure’s length, while the width is an average of the values measured at three locations along its length. The fine structures show collective motions and behave as part of an integrated self-contained magnetic flux tube. This is evident on two clearly visible fine structures showing periodic reversal of velocity sign, i.e., blue on top & red on bottom to red on top & blue on bottom. Thereafter, the previous original condition of velocity signs is restored, i.e., blue on top & red on bottom (Fig. 1A.2). It should be noted that blue and red are the signs of line-of-sight (LOS) components of respectively outward and downward motions, which are tangled with each other. This is the observational signature of torsional oscillations of the fine structured tube over an apparent surface.

RBEs/RREs have three instantaneous chromospheric motions, i.e., up-flows, swaying, and torsional motion^13^. The present observations differ from RBEs /RREs in two aspects: (i) an absence of flows along their length, and (ii) simultaneous red and blue-shifted emission. The adjacent red-blue shift pattern in the chromospheric line was also observed in newly discovered small-scale twisted flux tubes, although the time evolution of the Doppler shift pattern could not be revealed^14^.

## The method for the solar chromospheric observations and their analyses

As mentioned above this paper invokes solar chromospheric observational data to infer the presence of high-frequency Alfvén waves in the solar atmosphere. The observations are obtained with Crisp Imaging Spectropolarimeter (CRISP) on the ground-based Swedish 1-m Solar Telescope^15^,^16^. CRISP has a field-of-view of 60” × 60” (1” = 725 km) and a pixel scale of 0.0592”. The data is obtained with the cadence of 3.9 s on 10 June 2014 between 07:17 UT to 08:08 UT. The FOV of these observations was centered at (Xc, Yc) = (403”, −211”). It contains a pore and quiet-Sun region in the southward side of NOAA AR 12080. The adaptive optics (AO) system was running on SST and used the pore as a tracking point during the acquisition of the data. Nine Hα line positions are sampled in sequence, with eight images being collected at each of −1032, −774, −516, −258, 0 +258, +516, +774, +1032 mÅ w.r.t. the line core at 6562.8 Å before 36 frames were collected at each of −774 mÅ and the line core. After multi-object & multi-frame blind deconvolution (MOMFBD) reconstruction^17^, the desired data is obtained with resolution of 0.14”. All the observational data (cases) and their details are outlined in Supplementary Table 1 (see in Supplementary Material Information).

**Figure 1:**
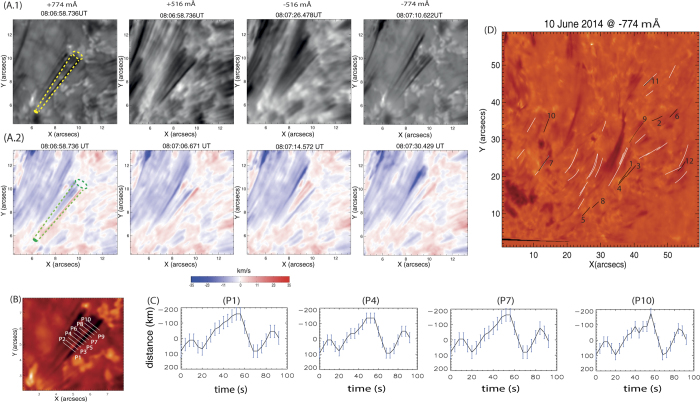
First observations of fine-structured solar magnetic flux tube showing high frequency torsional oscillations (<50 sec). The observations are made using the Crisp Imaging Spectropolarimeter (CRISP) on the ground-based Swedish 1-m Solar Telescope (cadence: 3.9 s with nine line positions across the Hα 6562.8 Å spectral line; image scale is 0.0592” per pixel corresponding to a spatial resolution of roughly 100 km) on 10 June 2014 between 07:17 UT to 08:08 UT. **A.1** (intensity) & **A.2** (Doppler) image sequence show a flux tube made by small-scale spicular-type fine structures anchored in the magnetic network and showing collective torsional motions on an apparent surface (see yellow & green expanding cylinders). (**B**) Displays the slits chosen across one such fine structure on the tube to measure the transverse motions in the projected 2-D plane as shown by fitted profiles in (**C**). (**D**) Displays a context image of SST/CRISP with all such observed flux tubes associated with clear torsional oscillations (black threads). The representative case presented in this figure is indicated by number ‘1’ in the context image.

The average line profile is defined by taking the average of the FOV and then the line profile is calculated using the pixels along the length of the event. Doppler shifts (Fig. 1A.2) are estimated w.r.t. the line center at Hα 6562.8 Å. Therefore, the velocities are estimated from a simple Doppler formula. We do not measure the full-width-at-half-maximum (FWHM) of the spectral-line as our event is observed in the Hα wings, which is sensitive to the temperature, opacity and velocity gradients, in addition to mass motions^18^. Moreover, we limit the spectral resolution by defining the line-positions to gain the required temporal resolution to observe high-frequency oscillations in the observed fast evolving chromospheric features.

## First direct observational detection of high-frequency Alfvén waves in the solar chromosphere

The transverse motion of one of these fine-structures on the tube is presented by choosing slits P_1_, P_2_, ..P_10_ at various spatial positions (Fig. 1‘B’). In a 2-D projected plane, it exhibits lateral transversal motions at various heights. In the time-distance plot at various locations (Fig. 1‘C’), the position of this dark structure and its displacement in time is estimated by locating the minimum intensity at each temporal step. The spatial position of the minimum intensity at each time step is estimated with sub-pixel accuracy using an FFT cross-correlation technique to disentangle the lateral fluctuations and the bulk motion of the fine structure, and fitting linearly its true transverse motions. Careful investigation shows almost negligible time-lag between the major peak and valley of the transverse motions of this structure at P_1_ and P_10_ (Fig. 1‘C’). A wavelet analysis of the transverse oscillations shows the presence of significant power associated with ~47 sec period (~21 mHz)^19^ (see Supplementary Material Information). Overall, this indicates the direct presence of long-wavelength high-frequency torsional waves that already moved to the top of the tube^20^. The periodic Doppler motions of the flux tube clearly show the torsional oscillations as pronounced on an apparent surface, which is the first direct observation of high-frequency (<50 sec) torsional Alfvén waves in the chromosphere. It should be noted that the first evidence of long-period (126–700 s) torsional Alfvén waves in terms of the oscillation of full-width-at-half-maximum (FWHM) of Hα spectral line had previously been reported above magnetic bright points^6^. The present observations reveal the first evidence of high-frequency torsional waves in numerous fine structured chromospheric flux-tubes (see Supplementary Table 1 in Supplementary Material Information) at larger scales along with more complete energy estimates using a stringent numerical model.

Direct observations of such high frequency torsional oscillations of the fine structured flux tubes in the chromosphere are difficult due to opacity, ongoing heating and cooling processes, and shallowness of the atmosphere^21^. Moreover, this requires high spatial and temporal resolution observations, which were made in the present case by SST/CRISP^16^,^17^ at various line positions around Hα 6562.8 Å. We observe twelve such cases of high frequency torsional oscillations on fine structured tubes. Their details and properties are given in Supplementary Table 1.

## The method for the stringent 3-D numerical simulation of high-frequency Alfvén waves

**Figure 2:**
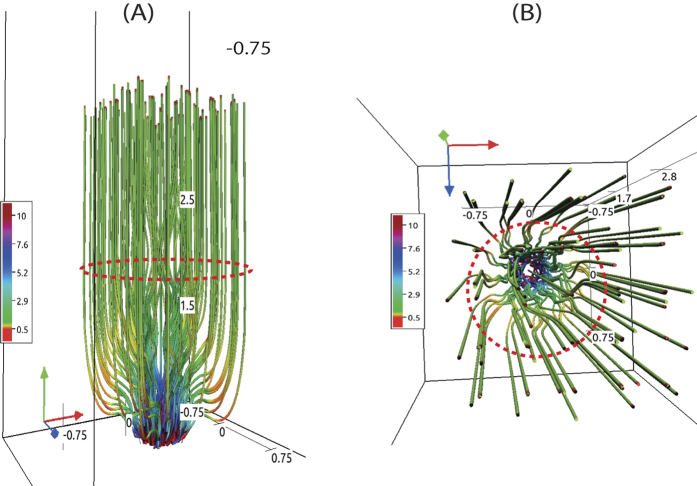
Transverse (‘**A**’) and Top-down (‘**B**’) views of a magnetic flux tube with representative magnetic field lines showing torsional motions. The height of the flux tube is 4 Mm, therefore, its upper boundary opens into the inner corona. The solar transition region is located at 2.1 Mm. The magnetic field at the foot-point is 121 Gauss (typical average strength of the quiet-Sun^22^). The tube is fanning out, and the dotted-red shell shows a schematic of an arbitrary apparent surface on which fine-structures may be subject to collective torsional motions.

A physical model of such localized solar structures, subject to the torsional oscillations (Fig. 1), is made by a simple but realistic magnetic flux tube using the FLASH code (Fig. 2). The model is an axis-symmetric tube rooted at the solar photosphere (B_o_ = 121 G)^22^, gravitationally stratified, and determined by realistic temperature distribution^23^. For the sake of simplicity, we model only the dynamics of a single enveloping tube similar in size to the observed fine structured flux tube. A concise description of 3-D magnetohydrodynamic (MHD) equations, initial conditions of the plasma and realistic magnetic field as well as their equilibrium in a gravitationally stratified tube, and numerical method and setup, are outlined in the literature^23^,^24^,^25^,^26^.

The flux tube, where magnetic and gas pressure are initially balanced keeping it in non-force free equilibrium, is then subjected to a perturbation in the azimuthal component of velocity by a periodic high-frequency driver. The form of velocity perturbation is given as,


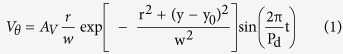


where A_v_ is the amplitude of the pulse, y_0_ = 500 km its vertical position, and w = 300 km its width. A_v_ is set to 150 km s^−1^, which results in an effective maximum velocity of about 2.4 km s^−1^. The period of the driver (P_d_) is taken as 50 s with wave perturbations generated at the top of the photosphere.

**Figure 3:**
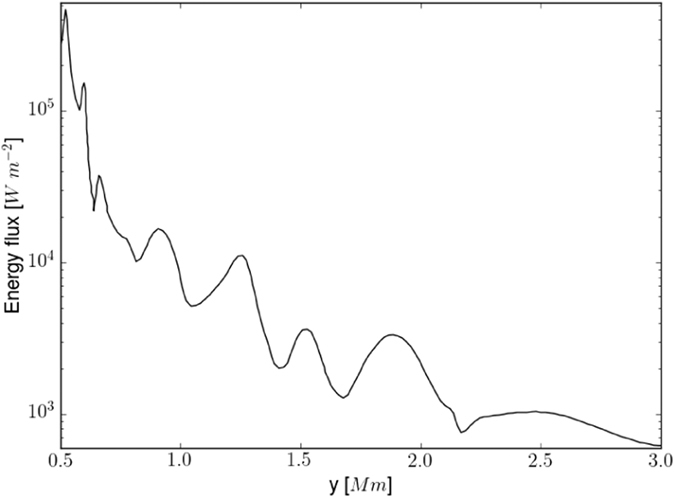
The energy flux of high-frequency torsional Alfvén waves along the tube. The wave has adequate energy of ~10^4^ W m^−2^ to heat the solar chromosphere, which is partially transported into the corona (~10^3 ^W m^−2^). The energy flux is estimated by averaging it over an area of the tube (−0.3 Mm < x < +0.3 Mm) around its central axis at each height.

In order to solve the 3-D MHD equations of the model^23^ numerically, we use the FLASH code^25^,^26^ with the third-order un-split Roe Riemann solver and the minmod slope limiter, as well as adaptive mesh refinement (AMR). We set the simulation box as (1.5, 1.5, 18) Mm^3^, and fix in time all plasma quantities to their equilibrium values at all six boundary surfaces. We use a static, non-uniform grid with a minimum (maximum) level of refinement set to 2 (5). Numerical results are displayed in Figs 2 and 3.

## Evolution of high-frequency Alfvén waves carrying substantial energy

The horizontal convective motions present in the quiet-Sun photosphere can generate Alfvén waves carrying significant amount of energy to heat the corona^27^,^28^. Such motions at the photosphere and chromosphere correlate well at the spatial scales of super-granular cells, therefore, indicating the transfer of energy seen as velocity fluctuations even up to the chromosphere^29^. Exclusively observed oscillating chromospheric flux tubes are visible even in the blue-wing (−774 mÅ) of Hα 6562.8 Å, which may form at least 200 km above the photosphere^30^. This indicates that the origin of transverse perturbations is somewhere at the top of the photosphere either by the transfer of gigantic velocity fluctuations or by impulsive magnetic reconnection^31^,^32^,^33^. Such impulses result in an azimuthal velocity perturbations (Equation 1) to excite the torsional Alfvén waves in the fine structured flux tubes.

Energy flux (W = 0.5 ρ V_A_ V^2^_θ_ in W m^−2^) is calculated using the estimated density (ρ), wave velocity amplitude (V_θ_), and local Alfvén velocity (V_A_) with height averaged over the horizontal spatial scales in the modeled flux-tube (−0.3 Mm < x < +0.3 Mm) by considering the tube as a whole. Therefore, the resultant velocity amplitude and thus derived energy flux at each height signify the net contribution of Alfvén waves excited in the tube. Energy flux oscillations result from the periodic driver which varies in time with the period, and its spatial averaging also contributes to the oscillations. As the energy flux is determined by V^2^_θ_, its averaging does not lead to a zero in the energy flux. The flux tube at its equilibrium is radially inhomogeneous. Therefore, Alfvén waves and their energy estimates weakly depend on a magnitude of equilibrium magnetic field. For a stronger magnetic field, the Alfvén speed may attain a larger value but it has little effect on the energy flux which depends on the equilibrium mass density and V^2^_θ_. The high-frequency Alfvén waves carry ~10^4 ^W m^−2^ energy in the chromosphere, and fulfill the huge requirement of energy there^34^. After partial reflection from the solar transition region, it still contains enough energy (~10^3 ^W m^−2^) to compensate the inner coronal energy losses^1^ (Fig. 3). This very basic analysis shows that the observed high-frequency torsional Alfvén waves can act as a substantial source to channel sufficient energy to heat the corona. Velocity fluctuations related to these Alfvén waves penetrate into the corona carrying associated energy (Fig. 3), while magnetic field perturbations are mostly concentrated up to the upper transition region and inner corona (Fig. 2). These structures are ubiquitous in the quiet-Sun chromosphere (Fig. 1). For the stronger magnetic field at the tube’s foot-point, the nature of the wave is the same. However, it may pump slightly more energy to the overlying atmosphere^20^.

The structuring of the plasma and magnetic field along the observed flux tube may lead to an amplitude variation as is evident in the standard-deviation (SDEV) measurement of the time-distance profile of transverse motions at its different locations (see Supplementary Material Information. This amplitude variation may further introduce a variation in energy flux. This is in qualitative agreement with the calculation of energy flux at different heights of the modeled flux tube (Fig. 3).

During MHD wave evolution in realistic model flux tube coupling the solar photosphere to the corona, various other important physical processes are evident. There is a generation of vertical plasma flows due to pondermotive forces under non-linear conditions. However, these up and down flows of the plasma are mostly trapped in the solar atmosphere and do not launch the solar wind unless the escape velocity is achieved. Another effect is the evolution of multiple concentric magnetic shells within the tube where Alfvén perturbations are pronounced with time creating complex velocity fields. Essentially, the velocity perturbations over different magnetic shells (or surfaces) are not in phase, which is a fundamental property of Alfvén waves in stratified inhomogeneous tubes.

The frequency range of the observed torsional Alfvén waves in various magnetic flux tubes lies between 12–42 mHz (Supplementary Table 1). Eight of these observed structures, associated with strong amplitudes, exhibit one full period of the traversal oscillations, while three show the half-period of the oscillations and disappear quickly (Fig. 1 and Supplementary Table 1). Our theoretical model depicts the wave propagation in Eulerian formalism and shows a clear evolution of the torsional oscillations. The observed chromospheric structures are the fast evolving features carrying similar high-frequency oscillations, but they fade away quickly. The major event presented in the paper (Fig. 1) exhibits almost two cycles of the oscillations vis-à-vis matching with the model. However, many of these incompressible waves, having large-amplitude, may undergo mode-conversion to compressible waves in a non-linear regime in the presence of a pondermotive force due to total magnetic pressure variations and may dissipate their energy quickly^35^. The fading of the oscillatory tubes may also be due to the strong velocity gradient at their edges and thereafter evolution of the Kelvin-Helmholtz instability^36^. It should be noted that these fast waves also seem to be associated with long wavelengths compared to the length of the tube itself. This is the reason why every tube shows torsional motion almost as a whole. These high-frequency torsional Alfvén waves are detected directly in such localized small-scale flux tubes.

Strongly homogeneous tubes in the chromosphere may be subject to coupling of the kink modes to the surface Alfvén waves. However, this may take a longer time compared to the dynamics of the observed structures here, while a discontinuity layer in the tube’s boundary is established with sufficient thickness^37^,^38^. Keeping in view the complexity (e.g., twist) in flux tube, the axis-symmetric sausage mode signature may also contribute to the Doppler velocity variations^39^. But, there is no evidence of an initial twist in the observed fine-structured tubes. Therefore, we rule out these possibilities in the present context. In conclusion, the SST/CRISP field-of-view of 60” × 60” (1” = 725 km) detects at least 40 oscillating flux tubes in the chromosphere. They seem to be ubiquitous in various regions of the Sun’s chromosphere^40^ indicating the presence of substantial energy sources beneath the corona. Torsional waves through many of these flux tubes (‘12’) carry sufficient energy up to the TR/inner corona, which may be potentially used in heating the localized atmosphere and energizing the supersonic wind by its dissipation due to energy cascade at smaller spatial scales^41^. These oscillating chromospheric structures will likely be detected in abundant measure with ultra high resolution observations with the next generation solar telescopes, e.g., 2-m National Large Indian Telescope (NLST), 4-m DKIST, European Solar Telescope (EST), 8-m Chinese Giant Telescope etc, and new information will further enlighten our understanding on their dynamics and potential role in the solar atmosphere^42^,^43^,^44^.

## Additional Information

**How to cite this article:** Srivastava, A. K. *et al*. High-frequency torsional Alfvén waves as an energy source for coronal heating. *Sci. Rep.* **7**, 43147; doi: 10.1038/srep43147 (2017).

**Publisher's note:** Springer Nature remains neutral with regard to jurisdictional claims in published maps and institutional affiliations.

## Supplementary Material

Supplementary Information

Supplementary Online M1

Supplementary Online M2

Supplementary Online M3

Supplementary Online M4

Supplementary Online M5

Supplementary Online M6

Supplementary Online M7

Supplementary Table Online Movie Case 1

Supplementary Table Online Movie Case 2

Supplementary Table Online Movie Case 3

Supplementary Table Online Movie Case 4

Supplementary Table Online Movie Case 5

Supplementary Table Online Movie Case 6

Supplementary Table Online Movie Case 7

Supplementary Table Online Movie Case 8

Supplementary Table Online Movie Case 9

Supplementary Table Online Movie Case 10

Supplementary Table Online Movie Case 11

Supplementary Table Online Movie Case 12
