# Supplementary material for: High-frequency torsional Alfvén waves as an energy source for coronal heating
Source: Sci Rep. 2017 Mar 3;7:43147. doi: 10.1038/srep43147 (PMC5335648; doi:10.1038/srep43147)
Supplement: Supplementary Information [file srep43147-s1.doc]

**Scientific Reports - NPG**

**High-frequency torsional Alfvén waves as an energy source for coronal heating**

**Abhishek Kumar Srivastava^1,*^, Juie Shetye^2^, Krzysztof Murawski^3^, John Gerard Doyle^2^, Marco Stangalini^4^, Eamon Scullion^5^, Tom Ray^6^, Dariusz Patryk Wójcik^3^, Bhola N. Dwivedi^1^**

**^1^Department of Physics, Indian Institute of Technology (BHU), Varanasi-221005, India. ^2^Armagh Observatory, College Hill, Armagh, BT61 9DG, N. Ireland. ^3^Group of Astrophysics, Institute of Physics, UMCS, Lublin, Poland. ^4^INAF-OAR National Institute for Astrophysics, 00040, Monte Porzio Catone, RM, Italy. ^5^Department of Mathematics & Information Sciences, Northumbria University, Newcastle Upon Tyne, NE1 8ST, UK. ^6^Dublin Institute for Advanced Studies, 31 Fitzwilliam Place, Dublin 2, Ireland.**

***Correspondence to asrivastava.app@iitbhu.ac.in**

**Supplementary Materials :**

**[1] Wavelet_Plot1&2.eps :** Powers spectrum associated with the time series of transveral displacements in the main event (case 1)presented in the paper is computed using the software provided by Torrence & Compo (1998). The first figure shows the global power and associated period present in the time-series, while second one displays the power distribution for various Fourier periods over the entire time-duration.

**
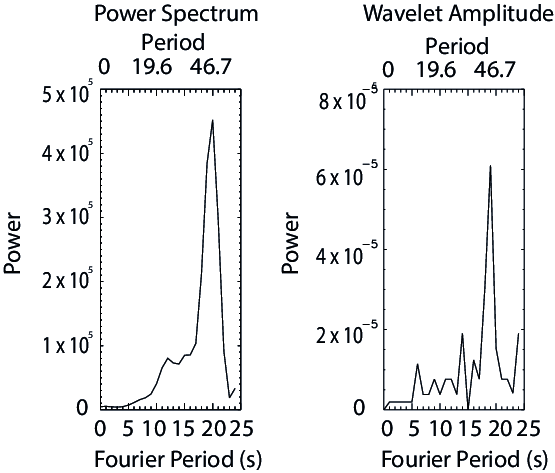
**

**
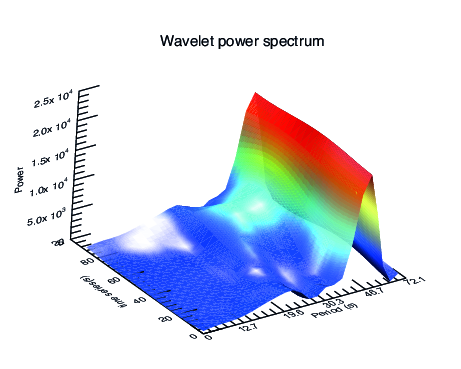
**

**[2] Supplementary table 1 :** The table provides a summary of 12 events as an oscillatory flux tubes reported in this paper. The 1-10 columns respectively outline case number, time (hh:mm:ss), duration of the dynamics (sec), context image, Doppler image, tube length (km), tube width (km), nature of the dynamics of the flux tube, period of oscillations (sec), amplitude of oscillations (km). The observations are obtained using Crisp Imaging Spectropolarimeter (CRISP) on the ground-based Swedish 1-m Solar Telescope. CRISP has a field-of-view of 60” x 60” (1” = 725 km) with a pixel scale of 0.0592”. The data is obtained with the cadence of 3.9 s with 09 line positions across the H_α_ 6562.8 Å spectral line on 10 June 2014 between 07:17 UT to 08:08 UT.

| **Case No** | **Time (hh:mm:ss UT)** | **Duration (s)** | **Context Image**  **(- 774 mÅ)** | **Doppler Image** | **Tube length (km)** | **Tube width (km)** | **Nature of the dynamics of the flux-tube** | **Period of the Oscillations (s)** | **Amplitude of the Oscillations (km/s)** |
| --- | --- | --- | --- | --- | --- | --- | --- | --- | --- |
| Case 1 | 08:06:46 | 80 | 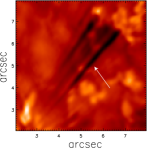 | 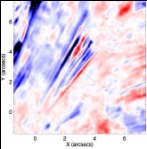 | 4000 | 129 | Torsional mode (Full Cycle). Lateral Displacement. | 48 | 150 |
| Case 2 | 07:57:16 | 120 | 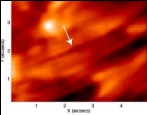 | 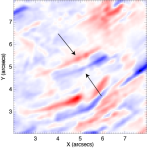 | 2500 | 129 | Torsional mode (Half Cycle). Lateral Displacement. | 80 | 85.5 |
| Case 3 | 08:20:36 | 40 | 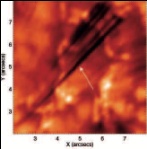 | 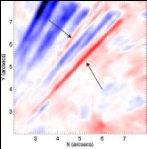 | 3000 | 182 | Torsional mode (Full Cycle). Lateral Displacement. | 24 | 210 |
| Case 4 | 07:49:01 | 84 | 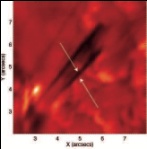 | 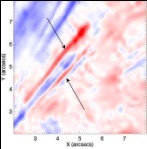 | 3000 | 182 | Torsional mode (Half Cycle). Lateral Displacement. | 28 | 420 |
| Case 5 | 07:54:46 | 80 | 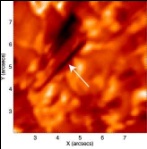 | 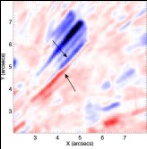 | 3800 | 126 | Torsional mode (Half Cycle). Transverse motion. | 56 | 171 |
| Case 6 | 08:03:44 | 92 | 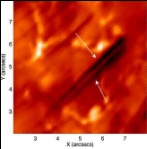 | 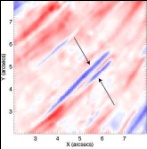 | 2500 | 154 | Torsional mode (Full Cycle). Lateral Displacement. Forking | 48 | 300 |
| Case 7 | 08:01:41 | 80 | 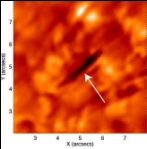 | 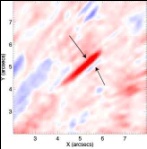 | 1800 | 214 | Torsional mode (Full Cycle). Lateral Displacement. | 40 | 514 |
| Case 8 | 07:54:46 | 48 | 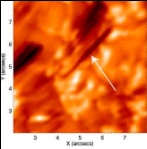 | 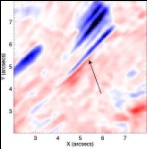 | 2500 | 171 | Torsional mode (Half Cycle). Lateral Displacement. Repeats. | 40 | 214 |
| Case 9 | 08:07:18 | 96 | 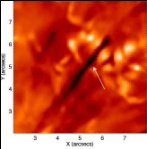 | 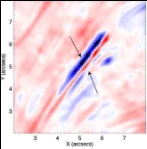 | 3000 | 214 | Torsional mode (Half Cycle). Lateral Displacement in one direction. | - | 214 |
| Case 10 | 08:20:18 | 80 | 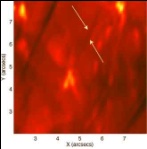 | 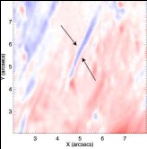 | 4800 | 129 | Torsional mode (Half Cycle). Lateral Displacement in one direction. | - | 256 |
| Case 11 | 07:35:41 | 16 | 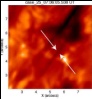 | 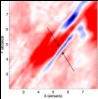 | 3800 | 175 | Torsional motion. | - | - |
| Case 12 | 07:49:01 | 80 | 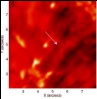 | 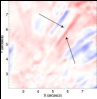 | 3200 | 126 | Torsional motion. | - | - |

**[3] STDDEV_Plot.eps : Amplitude variation of transverse oscillations along the observed flux tube (case 1).**

**
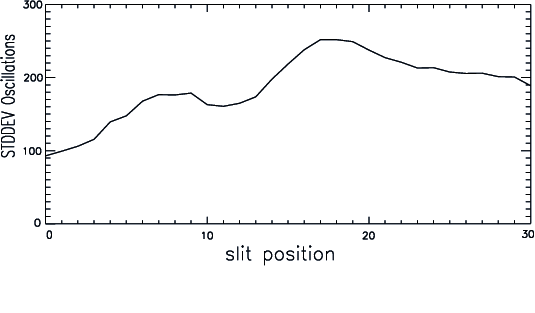
**

**[4] Various Movies :**

**Additional supplementary materials in form of movies of observational and numerical results are given below :**

**(i) Online_M1.gif :** Rotational motions of the flux tube (case 1) as evident by the tracked fine structures on the flux tube rooted in the quiet-Sun network and visible in H_α_ 6562.8 + 0.516 Å image sequence as observed by SST/CRISP.

**(ii) Online_M2.gif :** Rotational motion of the flux tube (case 1) is confirmed as Torsional oscillations. Two tracked fine structures of the tube show periodic reversal in velocity sign as evident in the Dopplergram sequence made by SST/CRISP observations.

**(iii) Online_M3.gif :** Motion of the modeled flux tube (transverse view).

**(iv) Online_M4.gif :** Motion of the modeled flux tube (Top-down view).

**(v) Online_M5.gif :** Vertical plasma flows in the modeled flux tube due to non-linear effects.

**(vi) Online_M6.mov :** Transverse cut of the modeled flux tube showing the evolution of magnetic shells.

**(vii) Online_M7.gif :**  Top-view of the velocity field in the modeled flux tube.

**(viii) Movies related to the observational events mentioned in the Table 1 as supplementary material.**

Table_Online_Movie_Case1.gif; Table_Online_Movie_Case2.gif; Table_Online_Movie_Case3.gif; Table_Online_Movie_Case4.gif; Table_Online_Movie_Case5.gif; Table_Online_Movie_Case6.gif; Table_Online_Movie_Case7.gif; Table_Online_Movie_Case8.gif; Table_Online_Movie_Case9.gif; Table_Online_Movie_Case10.gif Table_Online_Movie_Case11.gif Table_Online_Movie_Case12.gif
