# Supplementary figures and images for: High-frequency torsional Alfvén waves as an energy source for coronal heating
Source: Sci Rep. 2017 Mar 3;7:43147. doi: 10.1038/srep43147 (PMC5335648; doi:10.1038/srep43147)

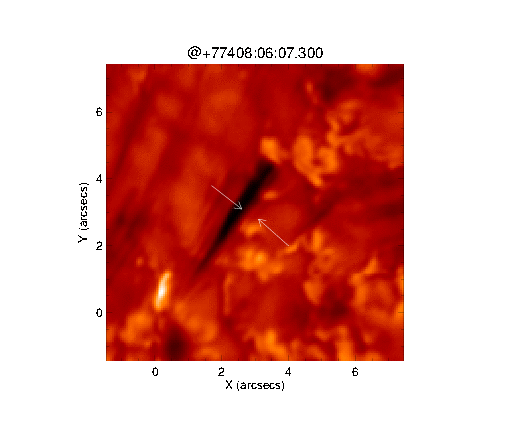

Supplement: Supplementary Online M1 [file srep43147-s2.gif]

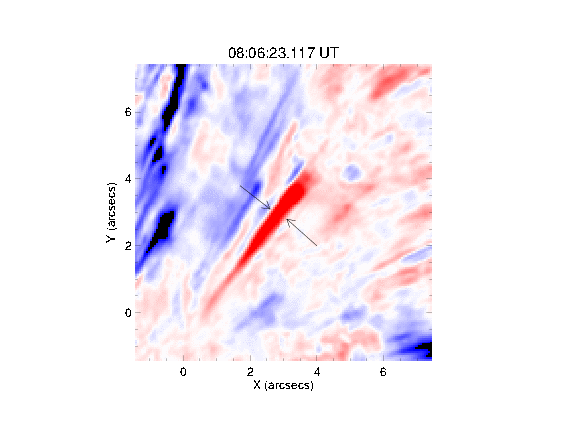

Supplement: Supplementary Online M2 [file srep43147-s3.gif]

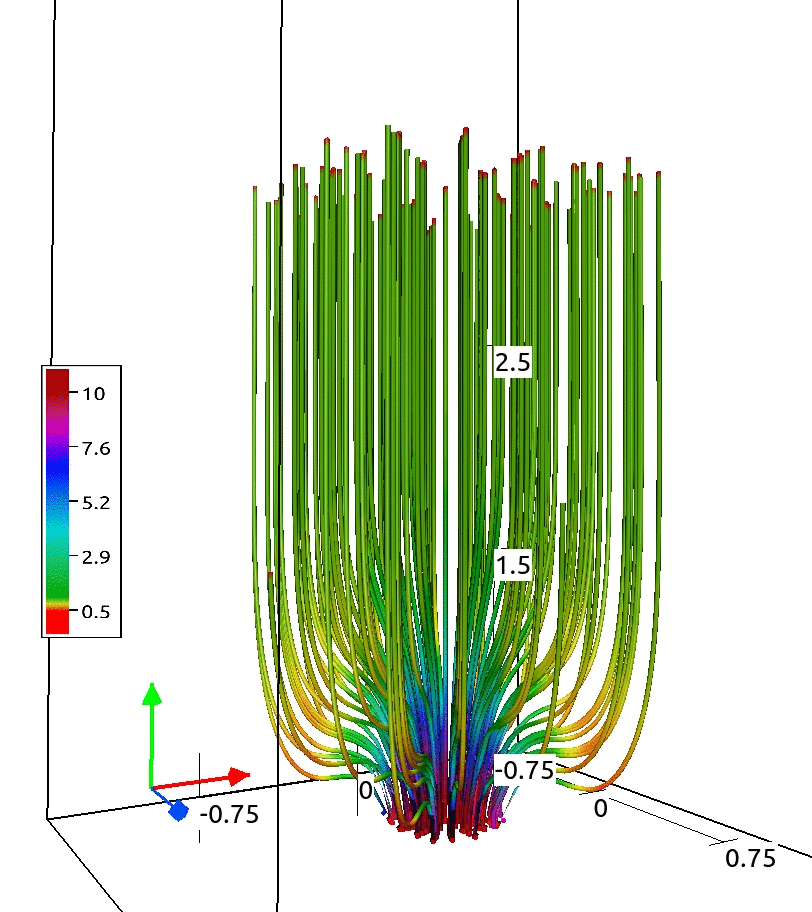

Supplement: Supplementary Online M3 [file srep43147-s4.gif]

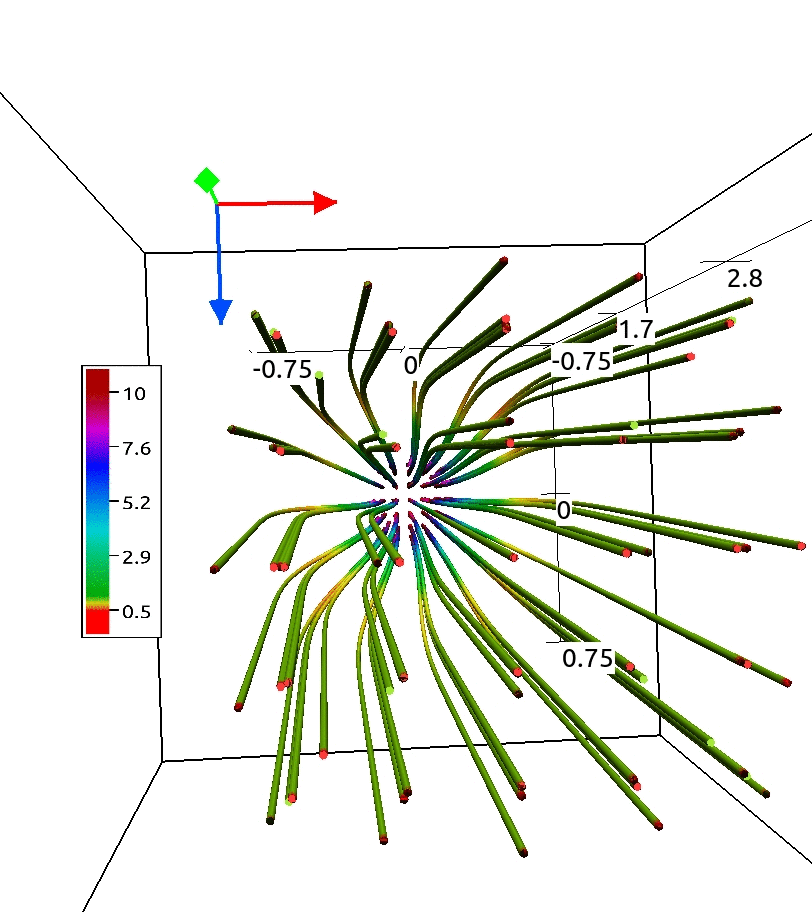

Supplement: Supplementary Online M4 [file srep43147-s5.gif]

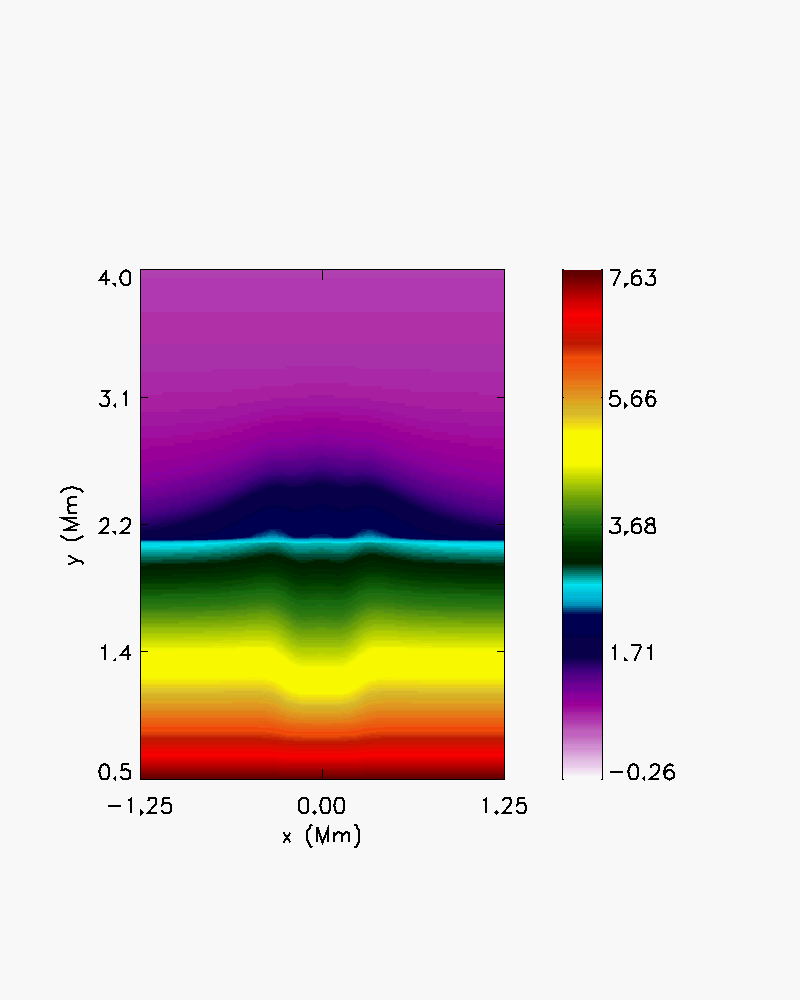

Supplement: Supplementary Online M5 [file srep43147-s6.gif]

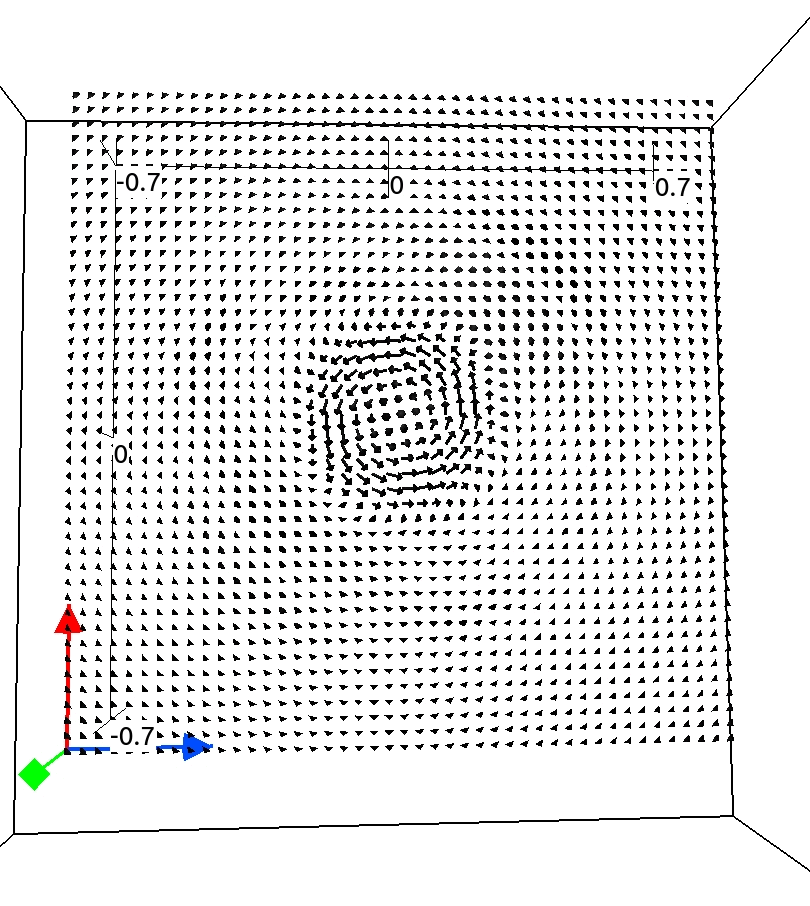

Supplement: Supplementary Online M7 [file srep43147-s8.gif]

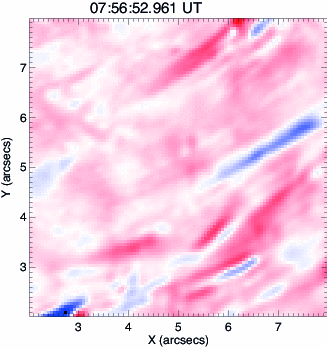

Supplement: Supplementary Table Online Movie Case 2 [file srep43147-s10.gif]

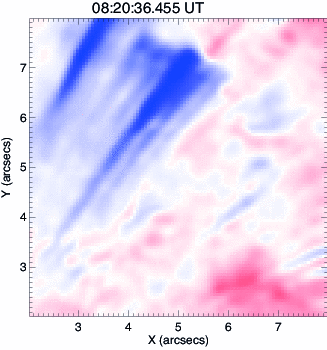

Supplement: Supplementary Table Online Movie Case 3 [file srep43147-s11.gif]

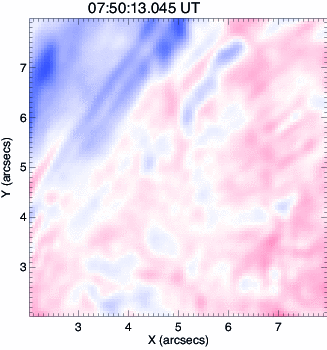

Supplement: Supplementary Table Online Movie Case 4 [file srep43147-s12.gif]

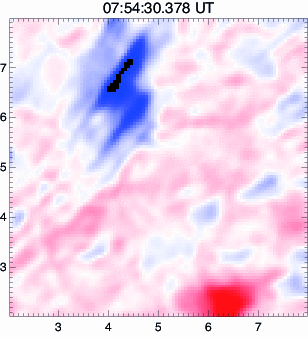

Supplement: Supplementary Table Online Movie Case 5 [file srep43147-s13.gif]

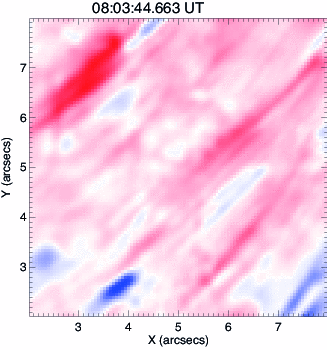

Supplement: Supplementary Table Online Movie Case 6 [file srep43147-s14.gif]

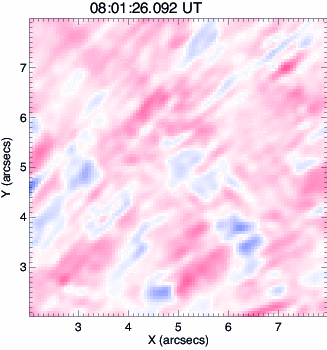

Supplement: Supplementary Table Online Movie Case 7 [file srep43147-s15.gif]

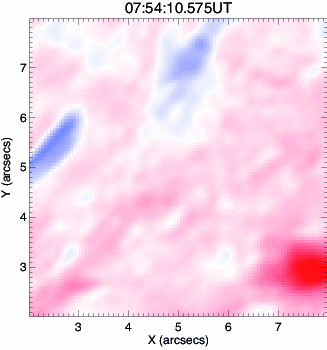

Supplement: Supplementary Table Online Movie Case 8 [file srep43147-s16.gif]

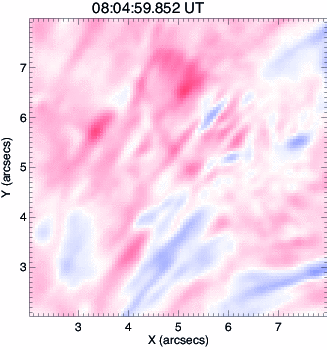

Supplement: Supplementary Table Online Movie Case 9 [file srep43147-s17.gif]

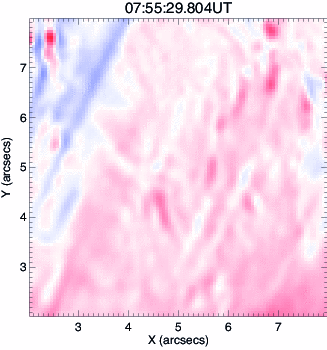

Supplement: Supplementary Table Online Movie Case 10 [file srep43147-s18.gif]

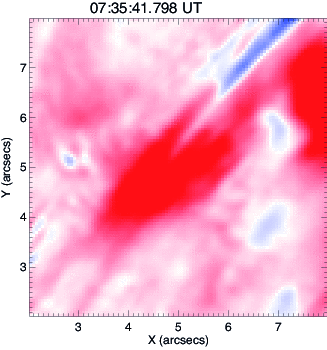

Supplement: Supplementary Table Online Movie Case 11 [file srep43147-s19.gif]

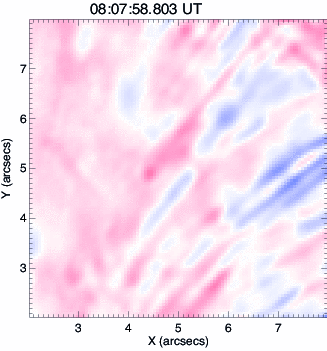

Supplement: Supplementary Table Online Movie Case 12 [file srep43147-s20.gif]
